# Effect on Quality of Life with Different Types of Obsessions in OCD Patients

**DOI:** 10.1192/bjo.2026.11190

**Published:** 2026-06-30

**Authors:** Sammar Fatima, Imtiaz Ahmad Dogar, Palvisha Sajid, Duaa Fatima, Asadullah Raoufy

**Affiliations:** Faisalabad Medical University, Faisalabad, Pakistan

## Abstract

**Aims:**

Obsessive–compulsive disorder (OCD) is a chronic psychiatric illness associated with marked functional impairment and reduced quality of life (QoL).Although the adverse impact of OCD on QoL is well established,local data is limited.In light of limited relevant local literature, we planned our research to build a scientific database related to effect on quality of life in OCD patients due to several different types of obsessions and to establish which type of obsessions are more prevalent and affect more.

**Methods:**

This cross-sectional study was conducted over a period of six months at the Department of Psychiatry and Behavioural Sciences, Allied Hospital II, Faisalabad, after approval from the Ethical Review Committee of Faisalabad Medical University. A total of 180 participants were recruited using probability consecutive sampling from the outpatient and inpatient services. Adults aged 18 years and above diagnosed with OCD according to DSM–5 criteria were included, while individuals with neurological disorders, significant cognitive impairment, acute psychiatric crises, or major comorbid psychiatric disorders were excluded. Written informed consent was obtained from all participants. Diagnosis and categorization of obsession types were established through structured clinical interviews based on DSM–5 criteria, and relevant data were recorded on a structured proforma. Data were analysed using SPSS version 25, with descriptive statistics calculated for demographic and clinical variables; differences in quality of life across obsession categories were assessed using analysis of variance (ANOVA), and simple linear regression analysis was performed to evaluate predictors of quality of life.

**Results:**

Of the 180 people who took part, 109 (60.6%) reported a poor quality of life, and 71 (39.4%) had a normal quality of life. There were 30 people in each group for each category of preoccupation. There was no statistically significant correlation between types of obsessions and quality of life (χ²=3.466, p=0.629). Nonetheless, QOL exhibited strong correlations with gender (p=0.038), employment status (p=0.022), disease duration (p=0.002), familial history (p=0.010), and OCD severity (p<0.001). A stronger link existed between worse quality of life and higher intensity and longer duration of illness.

**Conclusion:**

Patients with OCD have a significant deterioration in their quality of life. The specific type of preoccupation does not directly influence quality of life; rather, clinical considerations, especially the severity of OCD and the length of the illness, are pivotal. These results underscore the significance of prompt diagnosis, thorough management, and severity-oriented interventions to enhance the overall well-being of individuals with OCD.

